# Lentiviral Vector Delivery of Human Interleukin-7 (hIL-7) to Human Immune System (HIS) Mice Expands T Lymphocyte Populations

**DOI:** 10.1371/journal.pone.0012009

**Published:** 2010-08-06

**Authors:** Ryan M. O'Connell, Alejandro B. Balazs, Dinesh S. Rao, Christine Kivork, Lili Yang, David Baltimore

**Affiliations:** 1 Division of Biology, California Institute of Technology, Pasadena, California, United States of America; 2 Department of Pathology and Laboratory Medicine, David Geffen School of Medicine, University of California Los Angeles, Los Angeles, California, United States of America; New York University, United States of America

## Abstract

Genetically modified mice carrying engrafted human tissues provide useful models to study human cell biology in physiologically relevant contexts. However, there remain several obstacles limiting the compatibility of human cells within their mouse hosts. Among these is inadequate cross-reactvitiy between certain mouse cytokines and human cellular receptors, depriving the graft of important survival and growth signals. To circumvent this problem, we utilized a lentivirus-based delivery system to express physiologically relevant levels of human interleukin-7 (hIL-7) in Rag2-/-γc-/- mice following a single intravenous injection. hIL-7 promoted homeostatic proliferation of both adoptively transferred and endogenously generated T-cells in Rag2-/-γc-/- Human Immune System (HIS) mice. Interestingly, we found that hIL-7 increased T lymphocyte numbers in the spleens of HIV infected HIS mice without affecting viral load. Taken together, our study unveils a versatile approach to deliver human cytokines to HIS mice, to both improve engraftment and determine the impact of cytokines on human diseases.

## Introduction

The development of effective therapies against many of the most widespread human diseases is hampered by a lack of adequate model systems. Mice provide valuable in vivo models for basic research but are generally inadequate for the study of human-specific pathogens that infect cells of the human immune system. One promising solution to this dilemma has been the development of human-mouse chimeras that maintain the relatively low cost of small animal models while allowing for the study of human immune cells in a physiological setting. Human-mouse chimeras have been under development for close to 40 years, and significant progress has been made during this period[1]. However, the most successful of these models typically requires the implantation of multiple human embryonic tissues and are therefore labor intensive and expensive to create[2], [3].

Recently, two studies demonstrated that CD34+ human progenitor cells isolated from either umbilical cord blood (CB) or fetal liver could be injected into irradiated Rag2-/-γc-/- newborn mice resulting in the development of a human immune system (HIS)[4], [5]. Upon reaching adulthood, these mice develop both B and T lymphocytes that take residence in peripheral lymphoid organs including the spleen and lymph nodes. Similar engraftment has also been observed upon injecting CD34+ CB cells into NOD/scid γc-/- (NSG) recipient mice[6], [7]. These models demonstrate a limited, yet promising, functional response to immunization with tetanus toxoid or chicken ovalbumin resulting in some production of antigen specific antibodies by the graft[5], [6]. Further work has demonstrated that these models are susceptible to infection by human immunodeficiency virus (HIV), and can therefore be used for the study of this human pathogen[8], [9]. Despite this progress, HIS mice have significant limitations with respect to longevity of engraftment, production of myeloid cell populations, and the consistency of immune cell function in experimental replicates within a group[10]. Importantly, T cell populations in this model are slow to appear and are greatly outnumbered by B lymphocytes until the mice are greater than 6 months of age. These limitations necessitate the improvement of the HIS mouse model if it is to become a practical means of studying human immune responses or the pathology of human diseases.

One approach towards overcoming these deficiencies is to supplement human-mouse chimeras with cytokines to enhance engraftment and immune function. Previous studies have demonstrated improvements in human-mouse chimeras following cytokine therapy, typically promoting increased engraftment of certain cell types[7], [11], [12]. These studies have used both direct intravenous (iv) injection of recombinant cytokines and the creation of transgenic mouse strains to express specific factors. Both of these approaches have distinct disadvantages. iv injection leads to fluctuating levels of the delivered cytokine and is labor intensive. The transgenic approach is time consuming, especially when multiple strains are to be tested. We have developed an alternative cytokine delivery approach that can cheaply, easily, reliably and stably deliver precise doses of human cytokines to mice to improve the efficacy of human cytokine therapy, and provide greater versatility.

Human Interleukin 7 (hIL-7) is a hematopoietic growth factor implicated in the development of thymic T cells as well as lymphoid homeostasis and survival in the periphery[13], [14], [15], [16]. Upon binding to its cognate receptor IL-7Rα/CD127, signaling proceeds through the JAK-STAT pathway leading to activation of STAT5[17]. IL-7Rα signaling also regulates different BCL2 family members that are important regulators of cell survival[18]. Due to its profound impact on the homeostatic levels of T cells and lack of toxicity in vivo, IL-7 therapy is currently being used in clinical trials as a means to bolster T cell levels in lymphopenic individuals[19], [20], [21]. Because IL-7 is normally produced by stromal tissue and not immune cells[22], HIS mice are deficient in the human IL-7 signal. These features give exogenous delivery of hIL-7 the potential to improve the HIS mouse model.

In the present study, we have established a lentiviral vector-based platform for delivery of human cytokines to HIS mice with the long-term goal of improving human cell engraftment, diversity and functional capacity in this model. We have initially focused on delivering hIL-7 and found that super-physiological levels of hIL-7 could be produced and maintained for up to 6 months following a single injection of lentiviral vector encoding this cytokine. Adoptive transfer of human T lymphocytes into Rag2-/-γc-/- mice after they had received the hIL-7 expressing lentivector led to their homeostatic proliferation and expansion to higher levels compared to control conditions. Similar increases in T cell numbers were also observed in HIS mice that received the hIL-7 lentivector, and this correlated with higher expression of BCL2. Finally, an increase in T cell numbers in HIS mice receiving the IL-7 expressing lentiviral vector persisted in mice challenged with HIV, suggesting that IL-7 could be therapeutically beneficial in preserving CD4+ T cells during HIV infection.

## Results

### Increased survival of HIS mouse T cells following human IL-7 treatment *in vitro*

**Figure 1:**
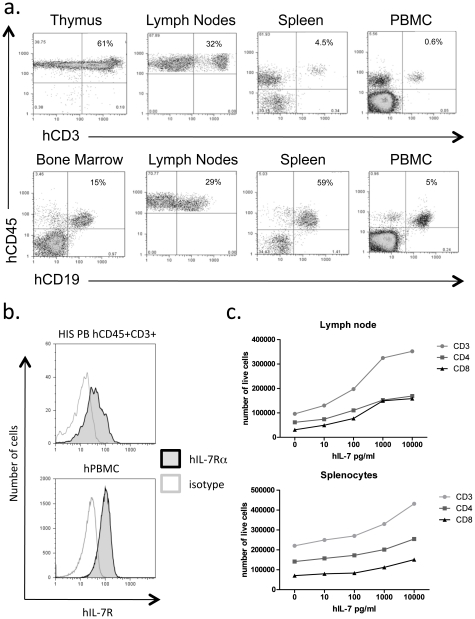
HIS mouse T cells express the IL-7Rα and exhibit increased viability in response to hIL-7 *in vitro*. **a.** Human immune cell populations were analyzed in lymphoid tissues from 20 week old HIS mice by flow cytometry to determine the extent of T and B cell engraftment. **b.** Peripheral blood from HIS mice or normal human donors was stained with either fluorophore-conjugated isotype control (clear histogram) or anti-hIL-7Ra antibody (grey histograms) and analyzed by flow cytometry. **c.** HIS mouse splenocytes (1×10^6^) or lymph nodes cells (5×10^5^) were cultured in increasing amounts of hIL-7 for 7 days. Cells were stained with antibodies against CD3, CD4 or CD8 as well as 7-AAD and analyzed by flow cytometry to quantify the number of live cells of each subtype.

We have established a HIS mouse model as described by Manz and coworkers[5], finding that injection of purified CD34+ CB cells into irradiated Rag2-/-γc-/- newborns can produce both B and T lymphocytes as these mice mature. B cells consistently dominate this model over the first 6 months, while CD3+ T lymphocytes are found at relatively low levels in the peripheral blood and spleens of these mice despite good numbers of developing T cells in the thymus (Figure 1a). Based upon these observations, we hypothesized that important human T cell growth factors might be missing from this system. Among such factors, IL-7 has been shown in both mice and humans to promote T cell survival and homeostatic proliferation[13], [14], [15], [16]. Furthermore, because this factor is produced by non-hematopoietic stromal cells it is not supplied by the engrafted human immune cells[22].

We first tested whether T cells that develop in HIS mice express the receptor for IL-7 by flow cytometry and observed expression levels similar to those found on normal human T cells in the peripheral blood (Figure 1b). Next, we treated both splenocytes (1×10^6^ RBC-depleted) and lymph node cells (5×10^5^) from HIS mice with increasing amounts of recombinant hIL-7 (replenished on day 4) and observed a dose dependent increase in the survival of both CD4+ and CD8+ T cells in vitro by day 7 based on 7-AAD staining (Figure 1c). This demonstrates that human T cells maturing in HIS mice are responsive to hIL-7.

### Systemic delivery of hIL-7 to Rag2-/-γc-/- mice using a lentiviral vector

**Figure 2:**
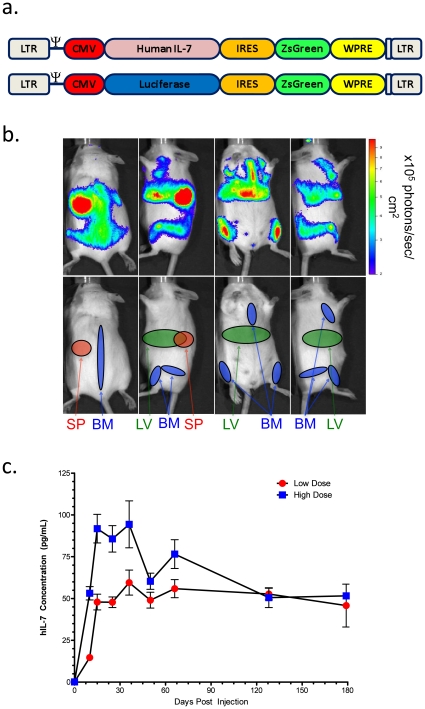
Delivery of hIL-7 to Rag2-/-γc-/- using a lentiviral vector. Schematic of the lentiviral vector used to deliver hIL-7 or luciferase. **b.** Expression of luciferase was assayed using Xenogen imaging two months after intravenous injection of Rag2-/-γc-/- mice with 2×10^8^ infectious units of luciferase expressing lentiviral vector. Localized expression from spleen (SP), bone marrow (BM), and liver (LV) are illustrated. **c.** Expression of hIL-7 was assayed for six months in the serum of Rag2-/-γc-/- mice following a single intravenous injection of either 1×10^8^ or 4×10^8^ infectious units of IL-7 expressing lentiviral vectors. Four mice were used per group, and the average and SEM are shown.

Lentiviral vectors have been used for long term systemic expression of genes in mice[23], and we therefore tested whether hIL-7 could be delivered to immunodeficient mice using such an approach. To this end we cloned the hIL-7 or luciferase (luc) genes into a lentiviral construct (Figure 2a), produced virus, and injected different amounts of hIL-7 or luc expressing virus intravenously into Rag2-/-γc-/- mice. Imaging of mice receiving the luc expressing lentivirus showed strong luciferase signal localized primarily to liver, bone marrow and spleen (Figure 2b) that was not present prior to transduction (Figure S1). In the serum we detected high levels of hIL-7 by ELISA, which were sustained for the entire six-month period of analysis (Figure 2c). Furthermore, the levels of hIL-7 were dependent upon the amount of lentivector injected for the first 2 months, indicating that the expression levels can be controlled. These data demonstrate that hIL-7 can be delivered to the immunodeficient mouse strains used as hosts for human immune system grafts and that expression is stable long-term. Additionally, intravenous administration of lentivirus causes infection of tissues known to be enriched in human immune cells, including the spleen and bone marrow[5], or which function as an endogenous source of IL-7, such as the liver[22].

### Lentiviral vector delivery of hIL-7 to Rag2-/-γc-/- mice promotes homeostatic proliferation of adoptively transferred human T cells

**Figure 3:**
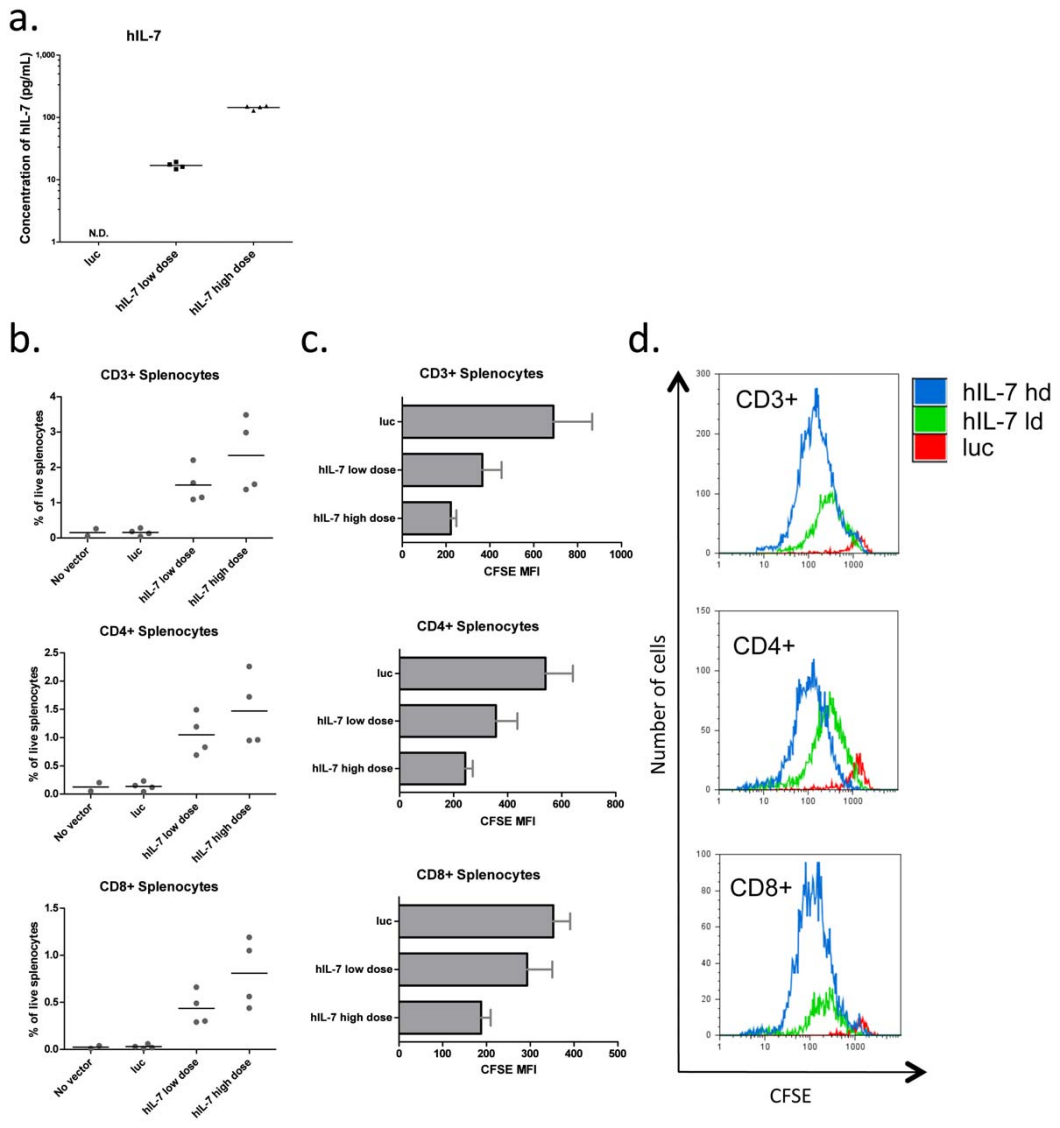
Lentiviral vector delivery of hIL-7 promotes homeostatic proliferation of adoptively transferred human T cells in Rag2-/-γc-/- mice. **a.** Serum concentrations of hIL-7 detected by ELISA three weeks after intravenous administration of 9×10^7^ or 1.7×10^8^ IU of lentivirus expressing either luciferase or hIL-7. **b.** The percentage of CD3+, CD4+ or CD8+ T cells of live splenocytes following one week post transfer of 2×10^7^ CFSE labeled human PBMCs into Rag2-/-γc-/- mice from A. **c.** Average mean fluorescence intensity (MFI) of CFSE measured by flow cytometry in T-cell subsets quantified in B. Four mice were used per group, and the average and SEM are shown. **d.** Representative histograms showing CFSE loss by CD3+, CD4+ or CD8+ adoptively transferred T cells from mice receiving the control vector, low dose hIL-7 or high dose hIL-7.

Having successfully produced stable levels of hIL-7 in Rag2-/-γc-/- mice following lentiviral vector injection, we next investigated whether hIL-7 produced by this method could impact human T cell survival as had been previously reported. Adult Rag2-/-γc-/- mice were injected with two different doses of the hIL-7 lentivector or control luc vector and hIL-7 expression was assayed in the mouse serum two weeks later. At this time point, we observed dose dependent levels of hIL-7 in mice receiving the hIL-7 producing lentiviral vector and no detectable hIL-7 in control mice (Figure 3a). Human peripheral blood mononuclear cells (PBMCs) were CFSE labeled and injected intravenously into these mice and allowed to expand for 7 days. Spleens were removed and T cell populations were quantified by flow cytometry. We observed a dose dependent increase in human CD3+ T cell populations in hIL-7 expressing mice as compared to mice expressing luciferase, and found that this increase occurred in both CD4+ and CD8+ T cell populations (Figure 3b and Figure S2). Because these cells were labeled with CFSE prior to injection we analyzed the intensity of dye labeling to determine the extent of their proliferation. Consistent with hIL-7 driving homeostatic proliferation of T cells, we saw a dose dependent decrease in CFSE intensity that inversely correlated with hIL-7 expression (Figures 3c and 3d). These data demonstrate that delivery of hIL-7 by a lentiviral vector can increase the number of T cells in Rag2-/-γ-/- mice by enhancing proliferation.

### Lentiviral delivery of human IL-7 to HIS mice expands human T cell populations in the peripheral blood

**Figure 4:**
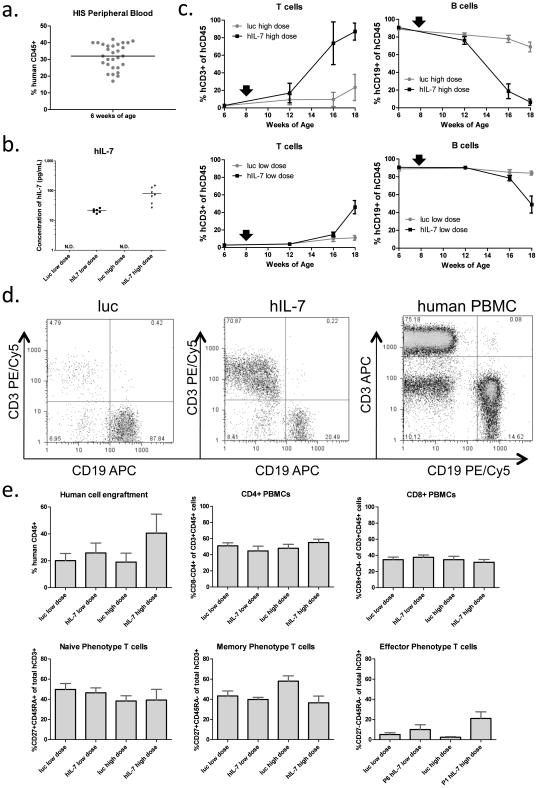
Lentiviral vector delivery of hIL-7 to HIS mice improves T cell levels in the peripheral blood. **a.** Total human CD45+ cell engraftment in peripheral blood of cohort of mice used in this experiment prior to separation into treatment groups. **b.** Serum concentrations of hIL-7 detected by ELISA at 18 weeks of age in mice receiving intravenous low (1×10^8^ IU) or high (5×10^8^ IU) dose lentivirus expressing either luciferase or hIL-7. **c.** HIS mice were injected with 1×10^8^ (low dose) or 5×10^8^ (high dose) hIL-7 or luciferase expressing lentiviral vectors at 8 weeks of age (indicated by black arrow). The percentages of CD3+ and CD19+ peripheral blood cells (as a percentage of total human CD45+ cells) were determined from 8 to 18 weeks of age by flow cytometry. 5–7 mice were used per group, and the average and SEM are shown.**d.** Proportion of CD3 and CD19 expressing cells in peripheral blood of a representative luciferase or hIL-7 expressing HIS mice at 18 weeks of age as compared to a normal human donor. **e.** For each mouse group, human cell engraftment was determined by calculating the percentage of human CD45+ cells of total CD45+ cells. The percentage of CD3+ CD4+ and CD3+CD8+ T cells in the peripheral blood was also determined. CD3+ naïve (CD27+CD45RA+), effector (CD27+CD45RA-) and memory (CD27-CD45RA-) phenotype T cells were quantified by flow cytometry.

Our adoptive transfer experiments indicated that hIL-7 could improve human T cell populations in chimeric mice, and suggested that this cytokine acts on the mature T cell populations found in PBMCs. As described above, CD34+ CB derived HIS mice have low levels of mature human T lymphocytes in their periphery. Thus, we tested the impact of lentivirus delivered hIL-7 on T cell numbers in HIS mice. A cohort of HIS mice was screened for human cell engraftment at 6 weeks of age (Figure 4a). Upon reaching 8 weeks of age, HIS mice were given high and low doses of the hIL-7 or luc control lentivectors, which again led to dose dependent expression of hIL-7 (Figure 4b). Of note, we did not detect any hIL-7 in engrafted HIS mice not receiving the hIL-7 expressing vector, demonstrating the inability of the graft to provide this cytokine. Peripheral blood B and T cell numbers were assessed periodically until 18 weeks of age (Figure 4c). Mice expressing hIL-7 showed a significant increase in the ratio of peripheral blood T cells to B cells when compared to luciferase expressing controls by 18 weeks of age (Figure S3). Interestingly, mice receiving the higher dose of lentivirus developed high levels of T-cells significantly faster than the low dose group (evident by 16 weeks of age) and reached a much higher level than the control and low dose groups by week 18. Importantly, blood from 18 week old hIL-7 expressing mice more closely resembled the T cell – B cell ratio observed in normal human blood than that of control mice (Figure 4d). Finally, we used flow cytometry to determine which T cell subsets were expanded by hIL-7 (Figure 4e). While hIL-7 led to marginally elevated human cell engraftment overall as compared to control mice, the relative proportions of CD4, CD8, naïve and memory phenotype T cells in the peripheral blood of hIL-7 expressing mice was largely unchanged by hIL-7, suggesting that all T cell populations were expanded. Of note, the high but not low dose group of hIL-7 mice had marginally elevated effecter T cell populations in the peripheral blood compared to control mice. Thus, hIL-7 can improve the overall T cell levels in the peripheral blood of HIS mice.

### IL-7 expands splenic lymphoid follicles and increases both T cell numbers and BCL2 expression

**Figure 5:**
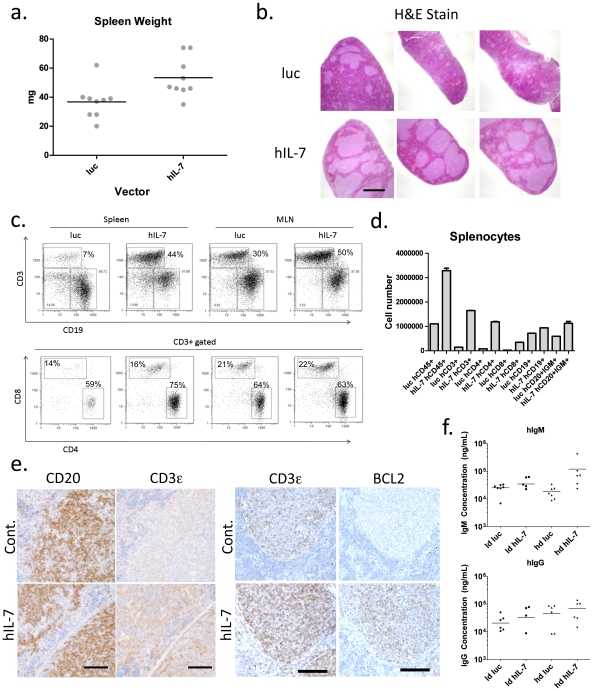
Lentiviral vector delivery of hIL-7 improves T cell levels in the spleens and lymph nodes of HIS mice, and increases BCL2 expression. Spleens were removed from hIL-7 (low dose group) or luciferase expressing mice and weighed. **b.** Splenic sections were H&E stained (scale bar  = 1 mm). **c.** Spleens and lymph nodes were processed into single cell suspensions and flow cytometry was used to analyze the percentage of human CD3+ versus CD19+ cells in the different groups. CD3+ cells were analyzed to quantify CD4+ and CD8+ subsets. **d.** Absolute cell numbers for the indicated lineages were determined using splenocytes from hIL-7 expressing or control mice. **e.** Serial splenic sections were stained with antibodies against human CD3 (scale bar  = 100 µm) or CD20 (scale bar  = 100 µm), and another set with CD3 (scale bar  = 100 µm) and BCL2 (scale bar  = 100 µm). **f.** Serum from both low and high dose hIL-7 expressing mice or luciferase controls was assayed to determine the concentrations of total IgM or total IgG.

We next examined whether hIL-7 impacted lymphoid tissues in HIS mice. Upon dissection, we observed modest splenomegaly in hIL-7 expressing mice compared to controls (Figure 5a), a finding that has been observed in people undergoing hIL-7 treatment in a clinical trial[21]. Hematoxylin and eosin staining on fixed spleen sections on demonstrated, on average, larger lymphoid follicles in hIL-7 treated mice (Figure 5b). Upon analyzing splenocytes by flow cytometry we observed a marked increase in the percentage of human T cells in the spleens and mesenteric lymph nodes of hIL-7 expressing mice but found that the CD4/CD8 ratio was not perturbed by IL-7 (Figure 5c). The absolute numbers of different cell types in the spleen were also determined and T cells were found to be elevated in IL-7 vs. control spleens (Figure 5d). Immunohistochemistry showed increases in the number of CD3+ T cells in splenic sections of hIL-7 expressing HIS mice as compared to controls, yet roughly equal densities of CD20+ B cells in the two groups (Figure 5e, left). Staining for the pro-survival factor BCL2 revealed elevated expression in spleens from hIL-7 expressing HIS mice, and overlapped with CD3+ T cells (Figure 5e, right) indicating a mechanism by which T-cells are expanded in the periphery of HIS mice expressing hIL-7. To assess the impact of improved T-cell engraftment on the humoral immune system, we assayed the serum levels of IgM and IgG and found an increase in total IgM, but little difference in total IgG concentrations in mice expressing hIL-7 (Figure 5f).

### IL-7 maintains high T cell levels in the spleen during HIV infection without boosting viral load

**Figure 6:**
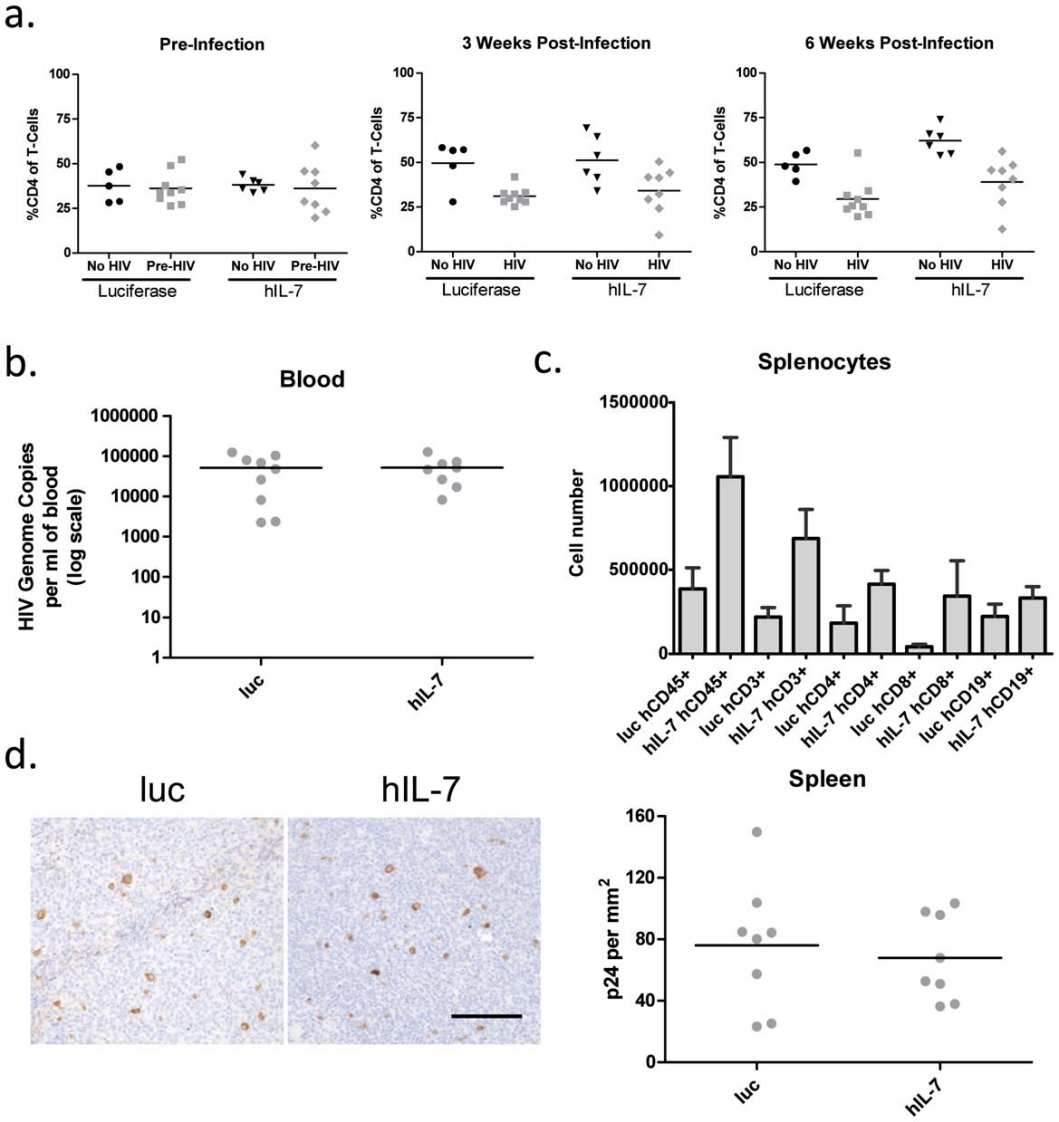
Higher CD3+ Splenic T cell numbers in HIV infected HIS mice expressing hIL-7. IL-7 or luciferase expressing HIS mice were infected with the HIV strain JR-CSF and peripheral blood was assayed by flow cytometry to determine the percentage of CD4+ of CD3+ human T cells. **b.** The HIV genome copy number in the peripheral blood plasma from the two groups of mice was quantified following 6 weeks of infection. **c.** The human CD45+, CD3+, CD4+, CD8+ and CD19+ splenocyte numbers were quantified by FACS 6 weeks after infection by HIV. **d.** Fixed Spleen sections from HIV infected luc and hIL-7 mice were stained for p24 (left, scale bar  = 100 µm). The number of p24 positive cells in a given area (1 mm^2^) of a lymphoid follicle was determined (right). Data represent 8–9 mice per group, and the average and SEM are shown.

An important feature of HIS mice is their ability to be infected by human-specific pathogens such as HIV, which depletes CD4+ T cells following infection[8]. Because IL-7 can increase the survival of T cells, we tested whether CD4+ T cells in HIS mice would be rescued by IL-7 following infection by HIV. To this end, we challenged hIL-7 expressing HIS mice with the CCR5-tropic HIV strain JRCSF at 18 weeks of age. Mice were bled and the fraction of CD4+ T cells (as a percentage of total CD3+ cells) in the peripheral blood was determined by flow cytometry. We observed a drop in CD4+ cells at both 3 and 6 weeks post-infection in both groups compared to uninfected animals (Figure 6a). Interestingly, quantitation of HIV genome copies in mouse serum six weeks post-infection by Amplicor revealed equivalent infection levels in HIS mice treated with either hIL-7 or control lentivirus (Figure 6b). Spleens were analyzed at six weeks and the number of total human cells (CD45+), T cells (CD3+, CD4+ and CD8+) and B cells (CD19+) in the spleens of infected mice were determined by FACS. Despite having equivalent systemic infection levels, hIL-7 treated mice exhibited elevated numbers of human T cells in their spleens compared to control mice (Figure 6c). Both groups had equivalent numbers of human splenic B cells. Fixed splenic sections were also stained for p24, and the number of HIV infected cells in a given area of lymphoid follicle was determined (Figure 6d). Like the blood, HIV infection of the spleen was similar between the groups. These data suggest that IL-7 can increase overall T lymphocyte numbers during HIV infection without affecting viral load.

## Discussion

The present study demonstrates that lentiviral vectors can be used to express cytokine genes in HIS mice following a single intravenous injection. This approach enabled hIL-7 to be expressed at consistent levels long-term, and improved human T lymphocyte numbers in the HIS model. Increases in T cell numbers occurred in all compartments analyzed, consistent with IL-7 functioning as a mediator of homeostatic proliferation. Additionally, the direct effects induced by IL-7 were entirely confined to the human graft, because the common γ chain required for IL-7R signaling is deficient in the host mouse strain.

Our approach resulted in hIL-7 serum concentrations higher than those found in healthy human adults, which ranges from 0.27 to 8.7 pg/ml (average 2.2 pg/ml), yet within the range of some HIV-positive lymphopenic individuals (which can be up to 70 pg/ml)[24]. Our experiments found that the impact of hIL-7 on human T cells was reduced in animals expressing lower concentrations of cytokine (10–20 pg/ml vs. 100 pg/ml hIL-7). This suggests that the HIS mouse model may require super-physiological levels of certain cytokines to induce meaningful changes in cellular compartments. The reasons for this are presently unclear, but it may reflect insufficient niche specific expression of hIL-7, which may be higher than systemic levels. Therefore, our expression platform might not be producing enough hIL-7 to reach such locations in sufficient quantities until we boost the overall systemic levels to super-physiological concentrations. Alternatively, other human cytokines not present in the HIS model might sensitize target cells to lower amounts of IL-7, but this also remains to be determined. It is also plausible that insufficient niche specific hIL-7 levels are the reason why a recent study did not observe differences in HIS mouse peripheral T cell levels following direct injection of recombinant hIL-7[25]. In fact, an earlier approach in which an Fc-IL-7 fusion protein was delivered via weekly injections led to increased T cell: B cell ratios in the spleens of humanized NOD-scid γc-/- mice[7]. This approach stabilized hIL-7 serum levels, resulting in a biological impact similar to that observed following constant hIL-7 production by our expression system in humanized Rag2-/-γc-/- mice.

We observed a direct correlation between vector dose and cytokine expression indicating that lentiviral based cytokine delivery can be easily adjusted to deliver a desired dose of cytokine. Transgene expression was also highest in the liver, bone marrow and spleen, consistent with an earlier study that used VSVG-pseudotyped lentivirus to deliver human factor IX to non-chimeric SCID mice[23]. This infection pattern exhibits significant overlap with the tissues and organs engrafted by the human immune cells such as spleen and bone marrow. Liver tissue was also efficiently targeted, mimicking one of the natural sites of IL-7 production in vivo. Given this overlap in infection and human cell engraftment patterns, it may also be possible to deliver molecules in addition to cytokines, such as transmembrane receptors, that require direct cell-to-cell contact to mediate their effects. Furthermore, because the technology to target lentiviral vectors to specific cell types in vivo is making substantial progress[26], delivering molecules to specific tissues in vivo may soon be feasible in the HIS model.

Recently, clinical trials have shown that hIL-7 can increase T cell numbers in people, indicating a promising therapeutic role for this cytokine[19], [20], [21]. Two of these studies examined HIV infected patients undergoing HAART therapy, resulting in low viral loads in these individuals and making it hard to assess the impact IL-7 on HIV replication. Our HIS mouse experiments suggest that IL-7 can improve T cell levels even in the absence of antiretroviral drugs, and do so without boosting HIV titers. This finding highlights the utility of HIS mice as models for human-specific disease.

IL-7 has been shown to function as an adjuvant in mice[27]. However, despite increased T cell numbers and lymphoid follicle size in the spleen, hIL-7 expressing HIS mice had only limited immune function similar to controls. Ovalbumin immunization resulted in modest antigen specific IgM responses and virtually no IgG production in both control as well as hIL-7 expressing HIS mice (Figure S4a). Additionally, JR-CSF infected hIL-7 or control mice did not develop any appreciable antibody responses to virus antigens as determined by western blotting (Figure S4b), further supporting our assessment that IL-7 expression alone was insufficient to restore normal immune function. Despite its inability to improve antigen specific immune responses, hIL-7 is likely an important piece of the puzzle given that T cells play a central role in immune responses to antigens. Consistent with this, we observed increased total serum IgM levels in hIL-7 expressing mice, suggesting that improved T-cell survival by hIL-7 resulted in increased B-cell output. Beyond immune function, IL-7 did not improve the mouse to mouse, or donor to donor, variability observed in the HIS model, suggesting that additional cytokines known to act on HSCs are likely necessary to impact overall human cell engraftment. Importantly, the modular nature of our system will permit the delivery of multiple cytokines at one time, and therefore future studies will investigate the effect of various cytokine combinations with the goal of enhancing human immune cell development and function in the HIS model.

## Materials and Methods

### Ethics Statement

All animal experiments were approved by the Institutional Animal Care and Use Committee (IACUC) and conducted in agreement with NIH policy. Animal experiments were conducted under California Institute of Technology IACUC protocols 1536-06T and 1547-08G.

### Mice

Rag2-/-γc-/- mice on a Balb/c genetic background were used for all mouse experiments. For adoptive transfer experiments, mice were injected with 2×10^7^ PBMCs (AllCells) that were first labeled with CFSE. HIS mice were created as described[5]. In brief, 1.5×10^5^ highly purified CD34+ CB stem cells (AllCells) were injected into the livers of irradiated (400 Rads) newborn pups within 24 hours of birth. Human cell engraftment was routinely determined by analyzing the ratio of human to mouse CD45+ cells in the peripheral blood at 6–8 weeks of age. Each experiment was performed using mice created from the same CD34+ cell donor population.

### Cell culture

T lymphocytes harvested from the spleen and lymph nodes of HIS mice were cultured in RPMI medium with 10% FBS and 1% Penicillin-Streptomycin. Cells were incubated in a humidified incubator (5% CO_2_) at 37 C. Recombinant hIL-7 (Ebiosciences) was added to the medium on day 0 and replenished on day 3 of culture, and cell viability was determined by staining for specific T cell surface markers (CD3, CD4 and CD8) and positivity for 7-AAD (eBioscience and Becton Dickinson).

### Lentiviral production and infection of HIS mice

Sequences encoding hIL-7 or luciferase were cloned into the pHAGE vector system. Experiments were performed using either pHAGE1 or pHAGE6 vector backbones containing identical transgenes driven from an internal CMV promoter for high and low doses respectively. Both vectors are third-generation, self-inactivating lentiviral vector backbones based on pHRST [28], [29]. Briefly, the StuI fragment of pHRST containing a complete viral genome was ligated into the pUC19 backbone to remove exogenous flanking genomic sequences. PCR-cloning was employed to introduce restriction sites flanking the promoter and transgene to facilitate subsequent cloning. Further modifications were made to pHAGE6 to remove extraneous viral sequences with no effect on virus function (A.B., to be published elsewhere). Lentivirus was produced by transient transfection of 293T cells with virus backbone and expression plasmids carrying helper proteins. Briefly, 30×10^6^ 293T cells were transfected with 40 ug of DNA using TransIT-293 reagent (Mirus Bio, Madison WI) according to manufacturer's instructions. Viral supernatants were collected beginning at 48 hours post-transfection and every 12 hours following for a total of 4 collections. Supernatants were subjected to ultracentrifugation in a Beckman SW28 rotor at 16,500rpm for 1.5 hours at 4C and pelleted virus was resuspended in growth media. Viruses were titered by standard means using flow cytometry of infected 293T cells to determine infectious units per mL. HIS mice were injected retro-orbitally with 200 uL of PBS containing the specified dose of virus per mouse. Following viral infection and integration, transcripts were initiated from an internal CMV promoter, carried the ZsGreen fluorescent protein downstream of an IRES element and contained a WPRE.

### Flow Cytometry

Blood was obtained via retro-orbital bleeding of anesthetized mice, or spleens and lymph nodes were removed from euthanized animals, homogenized, and passed through a 40 uM filter to create a single cell suspension. RBCs were lysed using RBC lysis solution, Fc receptors blocked using anti-mouse CD16/32 antibodies (eBioscience), and single cell suspensions were stained with specific antibodies using FACS buffer (1xPBS, 2% FBS, 0.1% BSA and 0.1% Sodium Azide). The following antibodies were used: anti-human CD3, CD4, CD8, CD19, CD45, CD127, CD27, CD45RA and IGM (eBioscience). Anti-mouse CD45.2 was also used to identify mouse white blood cells (eBioscience).

### Immunohistochemistry

Spleens were fixed in formaldehyde and embedded in paraffin by standard histological protocols. They were then sectioned and stained with H&E or immunohistochemistry was carried out with anti-human CD3, CD20 or BCL2. Anti-p24 antibodies were used to detect HIV. Stained sections were then visualized using an Olympus BX-41 light microscope and images captured and processed using SPOT imaging software. For quantification of p24 staining, ten 400X fields were counted for cells with definitive cytoplasmic staining. These were then presented as p24+ cells/mm^2^.

### HIV infections

HIV strain JRCSF (CCR5-tropic) was administered to HIS mice via i.p. injection of 2880 ng of p24 as quantified by ELISA in a volume of 200 µl. HIV genome copy numbers in the peripheral blood were determined using Amplicor (Roche).

### Ovalbumin Immunization

Balb/c or HIS mice previously receiving high dose luciferase or hIL-7 lentivirus were immunized with 100 ug Ovalbumin protein (Sigma) prepared with alum prior to IP injection. Serum samples were collected 1 week post immunization and frozen for future analysis. Mice were boosted 2 weeks after the initial immunization with a second injection of 100 ug of Ovalbumin in alum and serum was collected 1 weeks later (3 Weeks from initial immunization). Serums were serially diluted and applied to ELISA plates coated with Ova. Detection of Ova specific antibodies was achieved using anti-mouse IgM or IgG (Balb/C Samples) or anti-human IgM or IgG (HIS samples) HRP-conjugated antibodies. The absorbance at each dilution was measured and those in the linear range were used to calculate fold differences between the pre- and post-immunization samples.

### HIV-specific Western blot

Serum collected from mice previously infected with JR-CSF 7 weeks post-challenge was analyzed using the GS HIV-1 Western Blot system (BioRad) according to manufacturer's instructions. Briefly, serums were diluted and incubated with nitrocellulose strips containing pre-blotted HIV proteins prior to washing and enzymatic detection of bound human antibodies.

## Supporting Information

Figure S1 Luciferase imaging prior to intravenous administration of lentiviral vector. Expression of luciferase was assayed using Xenogen imaging prior to intravenous injection of Rag2-/-γc-/- mice with lentiviral vector expressing luciferase. The same representative mouse shown in Figure 2b is shown prior to transduction.
(2.29 MB TIF)

Figure S2 Lentiviral vector delivery of hIL-7 promotes homeostatic proliferation of adoptively transferred human T cells in Rag2-/-γc-/- mice. Rag2-/-γc-/- mice previously transduced with luciferase or hIL-7 expressing lentivirus were injected with 2×10^7^ CFSE labeled human PBMCs. One-week post transfer, the numbers of CD3+, CD4+ and CD8+ T cells were counted from spleens to determine the effect of hIL-7 during adoptive transfer.
(0.17 MB TIF)

Figure S3 Lentiviral vector delivery of hIL-7 to HIS mice improves T cell ratios in peripheral blood. HIS mice were injected with 1×10^8^ (low dose) or 5×10^8^ (high dose) hIL-7 or luciferase expressing lentiviral vectors at 8 weeks of age. The ratio of CD3+ to CD19+ cells detected in peripheral blood were determined from 8 to 18 weeks of age by flow cytometry. 5–7 mice were used per group, and the average and SEM are shown.
(0.45 MB TIF)

Figure S4 Assessment of antigen specific humoral responses in HIS mice expressing hIL-7. a. Wt Balb/c or HIS mice expressing luciferase or hIL-7 were immunized with ovalbumin protein and serums were subjected to ELISA following initial exposure (1 week) or post-boost (3 weeks) to quantify the fold increase in Ovalbumin specific IgM (top) or IgG (bottom) relative to pre-immune levels for each animal. Dashed lines are drawn at the level of no fold change. b. Serums from HIS mice expressing luciferase or hIL-7 infected with JR-CSF HIV for 6 weeks were subjected to western blot analysis to detect HIV specific antibody responses.
(3.98 MB TIF)
