# Supplementary figures and images for: Lentiviral Vector Delivery of Human Interleukin-7 (hIL-7) to Human Immune System (HIS) Mice Expands T Lymphocyte Populations
Source: PLoS One. 2010 Aug 6;5(8):e12009. doi: 10.1371/journal.pone.0012009 (PMC2917362; doi:10.1371/journal.pone.0012009)

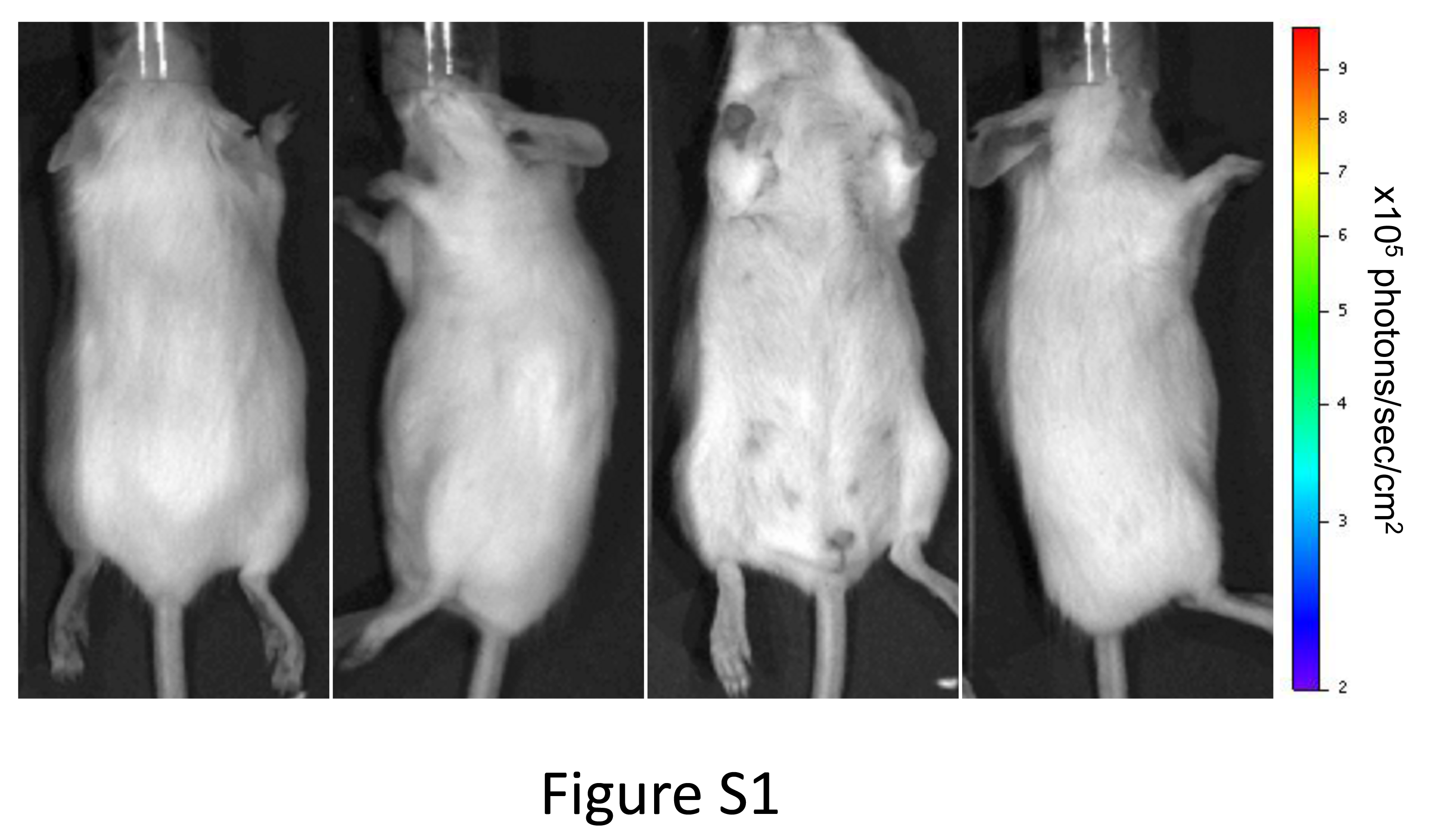

Supplement: Figure S1 — Luciferase imaging prior to intravenous administration of lentiviral vector. Expression of luciferase was assayed using Xenogen imaging prior to intravenous injection of Rag2-/-γc-/- mice with lentiviral vector expressing luciferase. The same representative mouse shown in Figure 2b is shown prior to transduction. (2.29 MB TIF) [file pone.0012009.s001.tif]

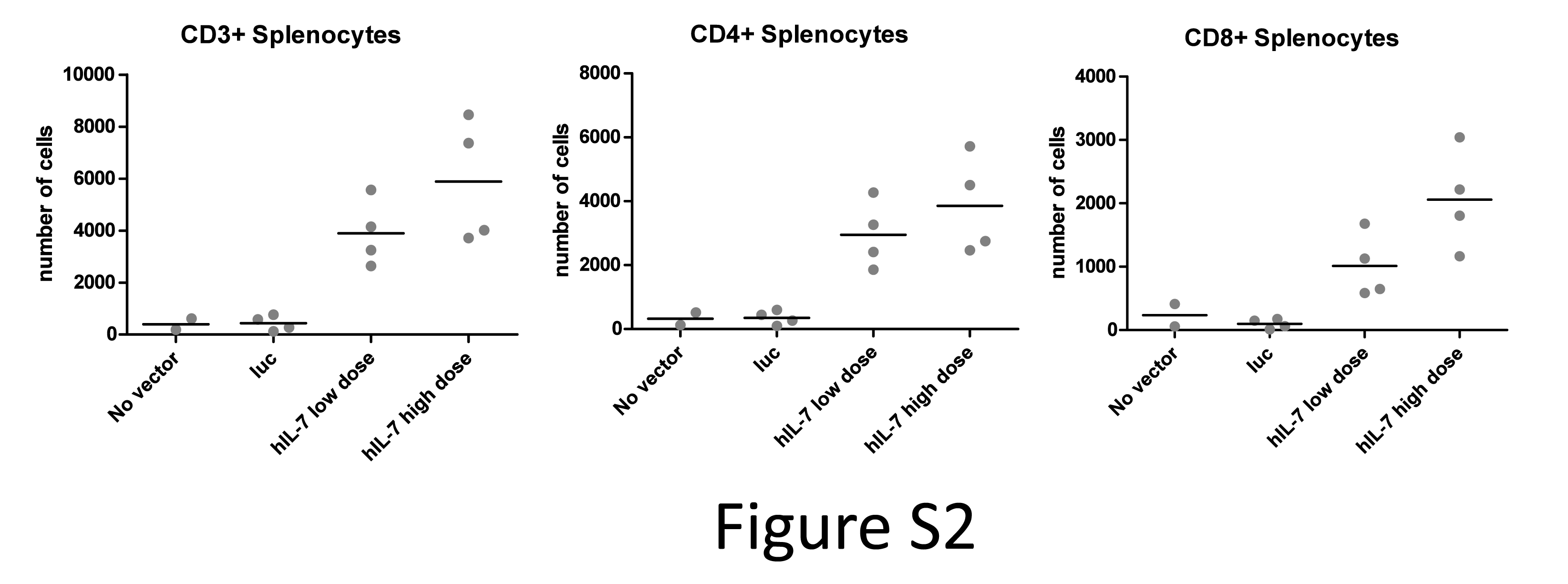

Supplement: Figure S2 — Lentiviral vector delivery of hIL-7 promotes homeostatic proliferation of adoptively transferred human T cells in Rag2-/-γc-/- mice. Rag2-/-γc-/- mice previously transduced with luciferase or hIL-7 expressing lentivirus were injected with 2×10^7^ CFSE labeled human PBMCs. One-week post transfer, the numbers of CD3+, CD4+ and CD8+ T cells were counted from spleens to determine the effect of hIL-7 during adoptive transfer. (0.17 MB TIF) [file pone.0012009.s002.tif]

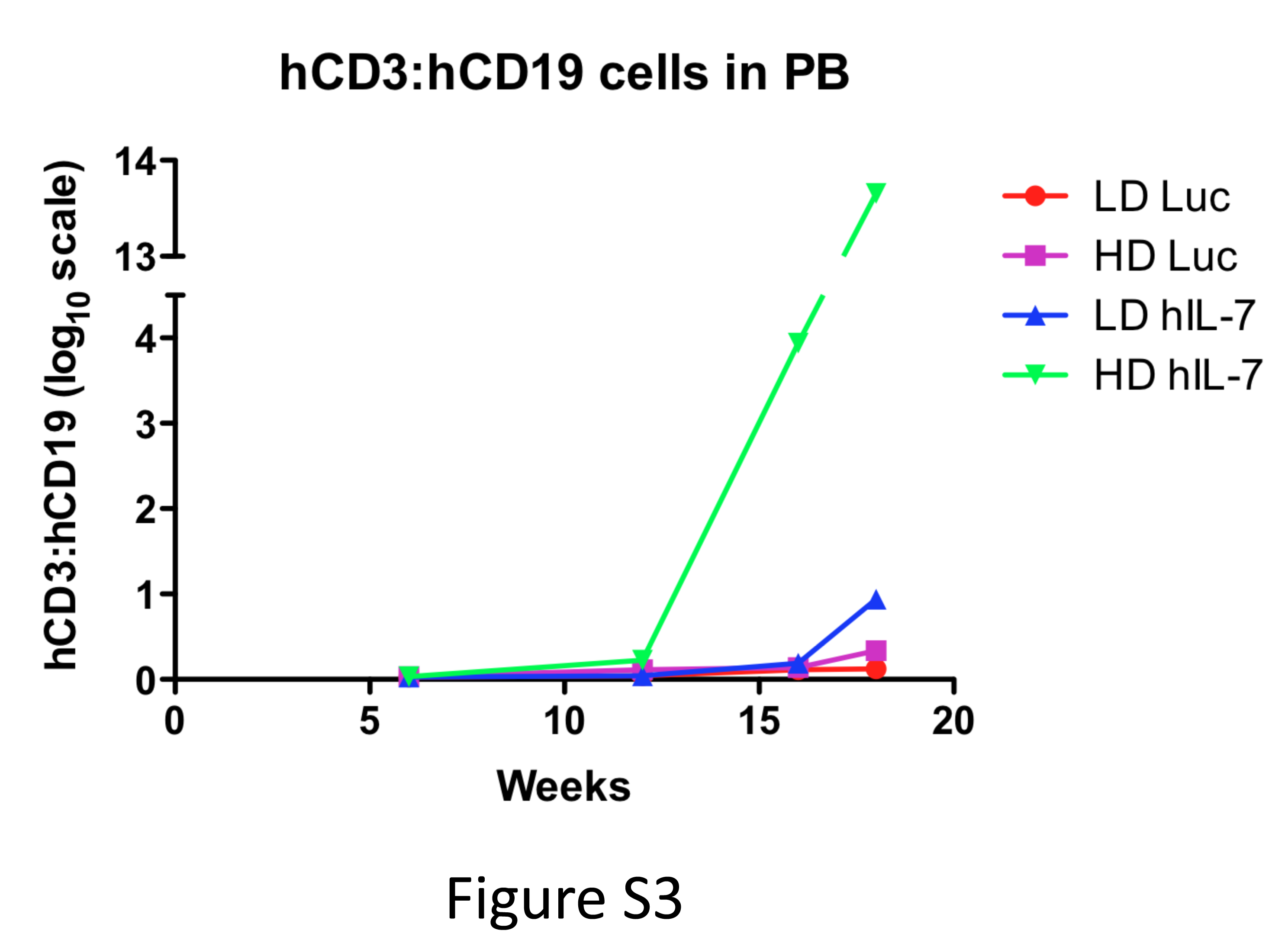

Supplement: Figure S3 — Lentiviral vector delivery of hIL-7 to HIS mice improves T cell ratios in peripheral blood. HIS mice were injected with 1×10^8^ (low dose) or 5×10^8^ (high dose) hIL-7 or luciferase expressing lentiviral vectors at 8 weeks of age. The ratio of CD3+ to CD19+ cells detected in peripheral blood were determined from 8 to 18 weeks of age by flow cytometry. 5–7 mice were used per group, and the average and SEM are shown. (0.45 MB TIF) [file pone.0012009.s003.tif]

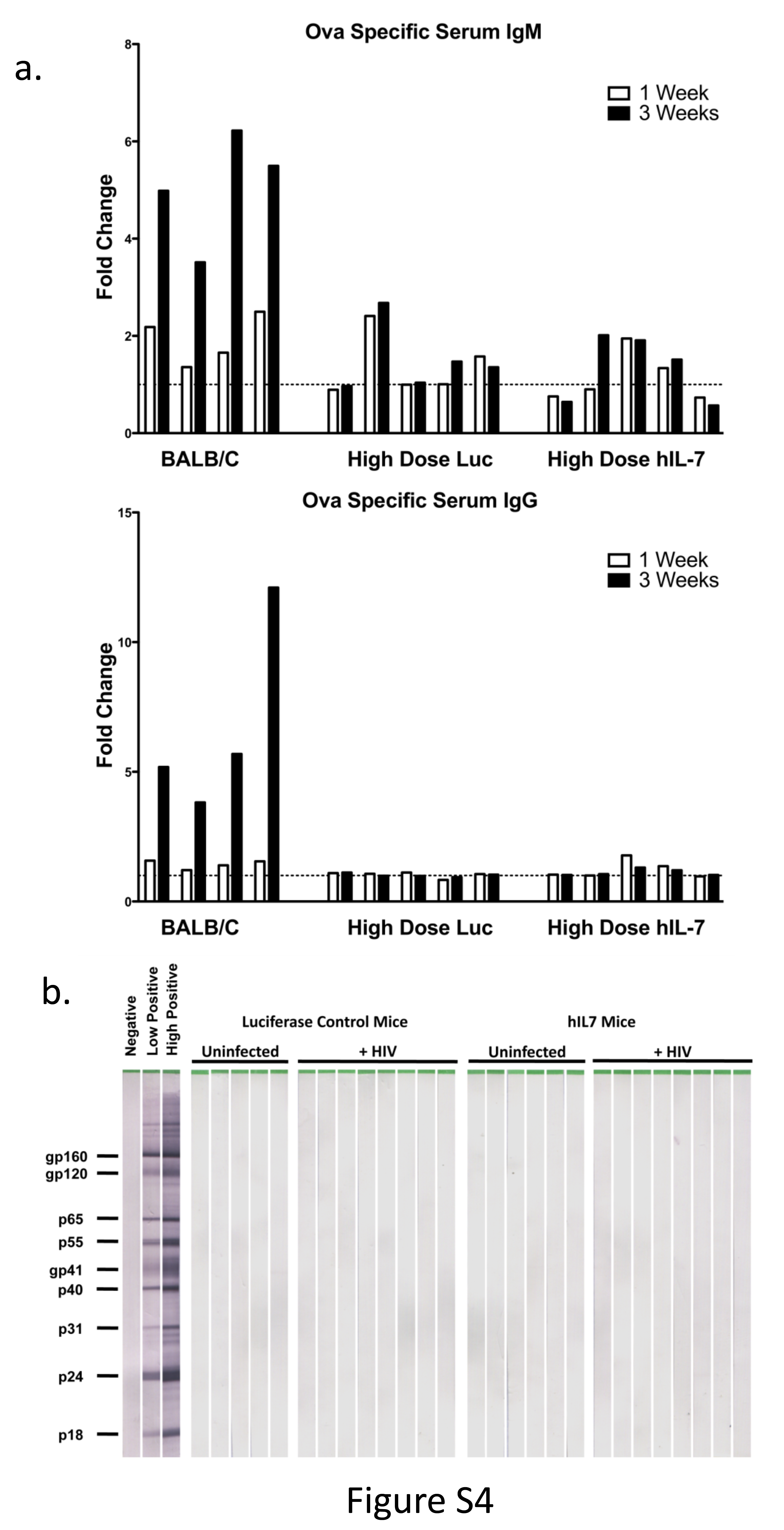

Supplement: Figure S4 — Assessment of antigen specific humoral responses in HIS mice expressing hIL-7. a. Wt Balb/c or HIS mice expressing luciferase or hIL-7 were immunized with ovalbumin protein and serums were subjected to ELISA following initial exposure (1 week) or post-boost (3 weeks) to quantify the fold increase in Ovalbumin specific IgM (top) or IgG (bottom) relative to pre-immune levels for each animal. Dashed lines are drawn at the level of no fold change. b. Serums from HIS mice expressing luciferase or hIL-7 infected with JR-CSF HIV for 6 weeks were subjected to western blot analysis to detect HIV specific antibody responses. (3.98 MB TIF) [file pone.0012009.s004.tif]
